# The current insights of mitochondrial hormesis in the occurrence and treatment of bone and cartilage degeneration

**DOI:** 10.1186/s40659-024-00494-1

**Published:** 2024-06-01

**Authors:** Wacili Da, Quan Chen, Bin Shen

**Affiliations:** Department of Orthopedic Surgery and Orthopedic Research Institute, West China Hospital, Sichuan University, Chengdu, Sichuan Province China

**Keywords:** Mitochondrial hormesis, Senescence, Osteoarthritis, Intervertebral disc degeneration, Osteoporosis

## Abstract

It is widely acknowledged that aging, mitochondrial dysfunction, and cellular phenotypic abnormalities are intricately associated with the degeneration of bone and cartilage. Consequently, gaining a comprehensive understanding of the regulatory patterns governing mitochondrial function and its underlying mechanisms holds promise for mitigating the progression of osteoarthritis, intervertebral disc degeneration, and osteoporosis. Mitochondrial hormesis, referred to as mitohormesis, represents a cellular adaptive stress response mechanism wherein mitochondria restore homeostasis and augment resistance capabilities against stimuli by generating reactive oxygen species (ROS), orchestrating unfolded protein reactions (UPRmt), inducing mitochondrial-derived peptides (MDP), instigating mitochondrial dynamic changes, and activating mitophagy, all prompted by low doses of stressors. The varying nature, intensity, and duration of stimulus sources elicit divergent degrees of mitochondrial stress responses, subsequently activating one or more signaling pathways to initiate mitohormesis. This review focuses specifically on the effector molecules and regulatory networks associated with mitohormesis, while also scrutinizing extant mechanisms of mitochondrial dysfunction contributing to bone and cartilage degeneration through oxidative stress damage. Additionally, it underscores the potential of mechanical stimulation, intermittent dietary restrictions, hypoxic preconditioning, and low-dose toxic compounds to trigger mitohormesis, thereby alleviating bone and cartilage degeneration.

## Background

Osteoarthritis (OA), intervertebral disc degeneration (IVDD), and osteoporosis (OP) stand out as significant degenerative diseases affecting bone and cartilage, with cellular senescence serving as the primary pathogenic factor. Extant research underscores that the molecular underpinnings of cellular senescence encompass telomere shortening, mitochondrial dysfunction, oxidative stress, DNA damage, a pervasive inflammatory response, radiation, and aberrant mechanical stimulation [1–5]. Mitochondrial dysfunction, identified as a pivotal contributor to age-related phenotypic anomalies, demonstrates a close association with the onset, progression, and prognosis of degenerative bone and cartilage diseases. OA, IVDD, and OP share analogous pathological features, including disturbances in energy metabolism, mitochondrial dysfunction, oxidative stress, inflammation, and abnormal protein aggregation. Targeted enhancement of mitochondrial function, spanning aspects such as metabolism, respiration, redox regulation, calcium signaling, biogenesis, dynamics, and quality control, has shown feasibility in numerous cellular and animal models. Consequently, the current research focus involves the prospect of alleviating or reversing the advancement of aging-related bone and cartilage degenerative diseases through the restoration or enhancement of mitochondrial quality.

In recent years, mitochondrial hormesis (mitohormesis), recognized as an adaptive protective mechanism of mitochondria, has garnered increasing attention within the biomedical research realm. This phenomenon specifically denotes an inherent adaptive stress response exhibited by mitochondria in the face of low doses of stressors that do not reach the damage threshold. Such a response serves to bolster mitochondrial function and augment stress resistance through the activation of pertinent protective molecules and signal transduction pathways [6, 7]. Mitohormesis engenders a spectrum of adaptive defense mechanisms within cells, encompassing enhancements in antioxidant capacity, fortification of the cellular protective response, augmentation of exogenous biological detoxification, and elevation of cellular metabolic flux [8, 9]. Consequently, this review endeavors to delve into the pathogenesis of mitochondrial dysfunction in aging-related OA, IVDD, and OP. Furthermore, it aims to scrutinize current research concerning the mitigation of bone and cartilage degeneration through the stimulation of mitochondrial hormesis and to delineate existing research gaps, laying the groundwork for future breakthroughs.

## Mitochondrial hormesis

Hormesis pertains to beneficial biological responses resulting from exposure to low continuous or moderate intermittent doses of stress, which may prove harmful at higher or chronic levels. When organisms encounter lower stress levels, it confers protection against more severe stress in subsequent exposures. The concept of mitohormesis, a term combining mitochondria and hormesis, was originally evidenced in simpler organisms [8]. Exposure to mild mitochondrial stressors has been shown to extend lifespan and mitigate aging-related diseases. This phenomenon has now been documented in mammals [10].

**Fig. 1:**
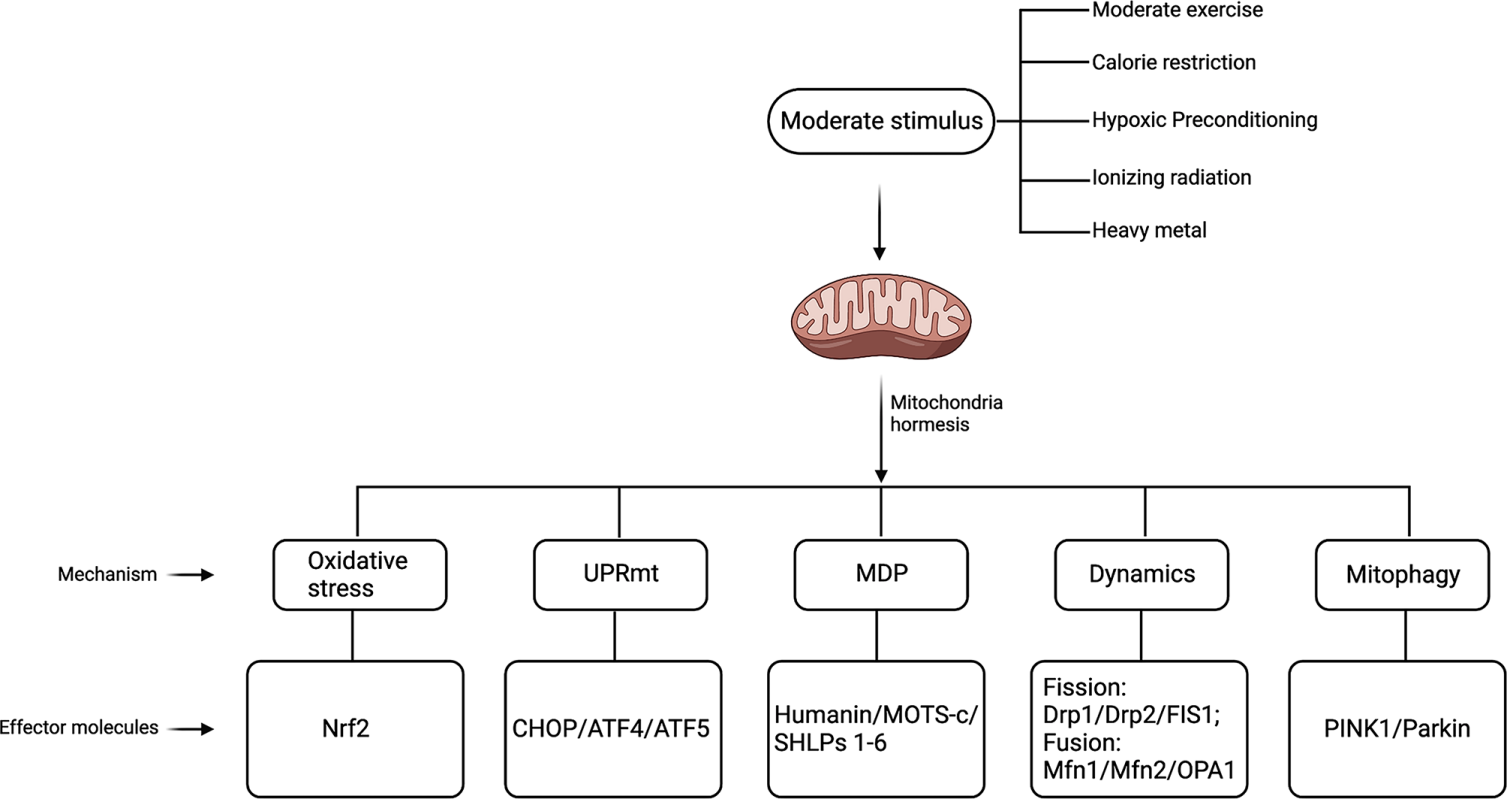
Schematic representation of the overall understanding of mitohormesis. ATF4: activating transcription factor 4, ATF5: activating transcription factor 5, CHOP: C/EBP homologous protein, Drp1/2: dynamin-related protein 1/2, FIS1: mitochondrial fission 1, Mfn1/2: mitofusin-1/2, MOTS-c: mitochondrial ORF of the 12 S rRNA Type-C, Nrf2: NF-E2 p45-related factor 2, OPA1: optic atrophy-1, PINK1: PTEN induced putative kinase 1, SHLPs 1–6: small humanin-like peptides 1–6

At present, there is a prevailing consensus that stressors capable of inducing mitohormesis primarily fall into distinct categories, including moderate exercise, dietary restrictions, hypoxic preconditioning, ionizing radiation, and exposure to low-toxic compounds [11–15]. Cells uphold mitochondrial homeostasis through various mechanisms, encompassing reactive oxygen species (ROS) regulation, unfolded protein reaction (UPRmt), mitochondrial-derived peptides (MDP), dynamic alterations in mitochondrial morphology (fission and fusion), and the process of mitophagy (Fig. 1). These intricate processes collectively serve to sustain cell survival and uphold biological efficacy [16–21].

## Oxidative stress

**Fig. 2:**
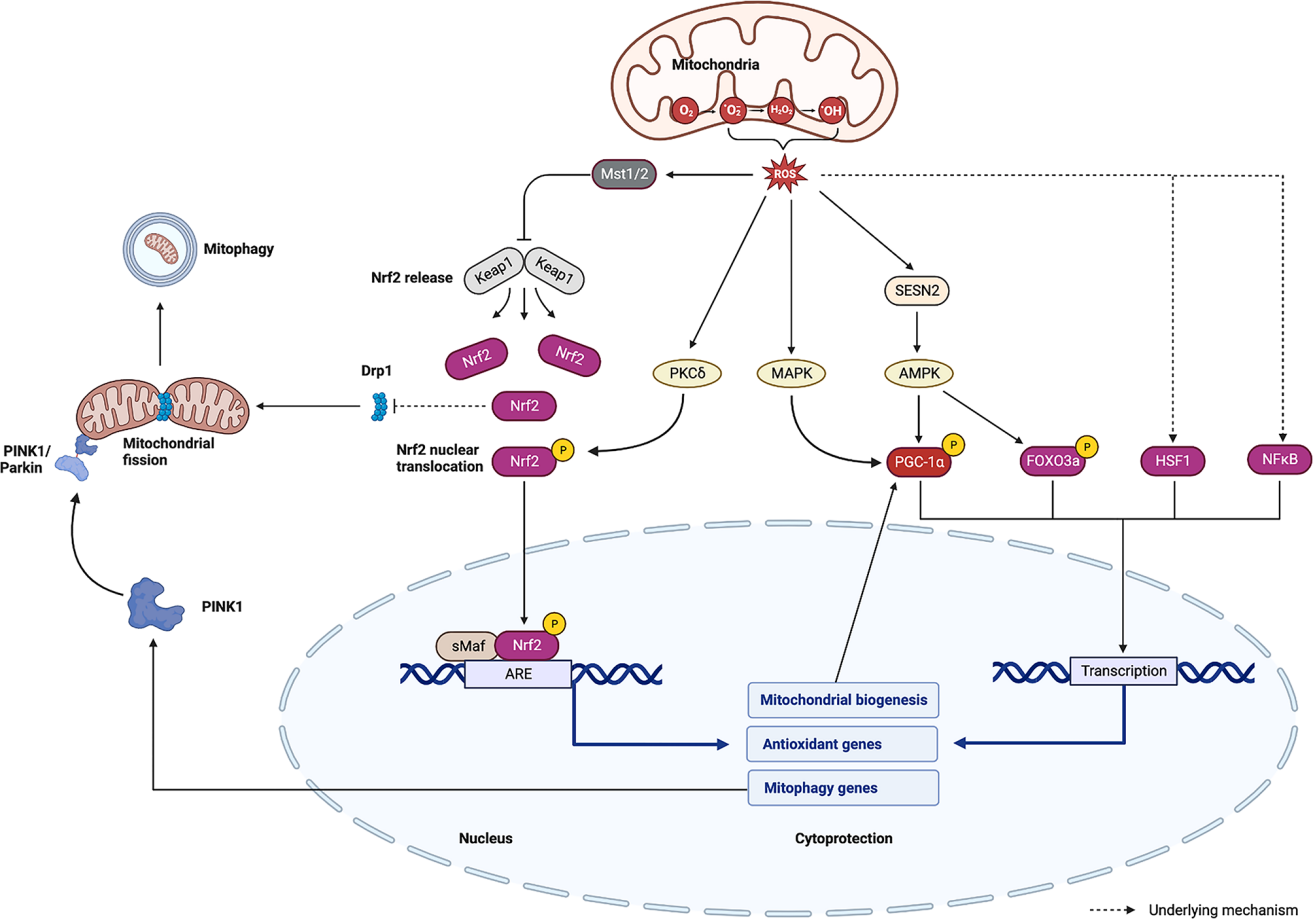
Oxidative stress signaling system of mitohormesis. There are synergistic and positive feedback regulation between oxidative stress and mitophagy. AKT: serine/threonine kinase proteins, AMPK: adenosine 5’-monophosphate (AMP)-activated protein kinase, ARE: antioxidant response element, FOXO3: forkhead box class O3a, HSF1: heat shock factor 1, Keap1: Kelch-like ECH-associated protein 1, MAPK: mitogen-activated protein kinase, Mst1/2: mammalian Ste20-like kinases 1/2, NFκB: nuclear factor kappa-B, PGC-1α: proliferator‐activated receptor gamma coactivator 1‐alpha, PKCδ: protein kinase Cδ, SESN2: Sestrin2, sMaf: small Maf

The ROS cascade stands as the principal signaling system for mitohormesis, as illustrated in Fig. 2. Within the mitochondrial oxidative phosphorylation electron transfer chain, electron spillovers (1-5%) give rise to ROS such as superoxide anions (O_2_•−), hydroxyl radicals (•OH), and hydrogen peroxide (H_2_O_2_) [22, 23]. Ionizing radiation predominantly generates ROS, including •OH, through the ionizing decomposition of water in tissues and cells [14]. ROS elicit a mitohormic effect by activating downstream transcription factors, fostering communication between mitochondria and the nucleus. Nuclear factor (erythroid-derived 2)-like 2 (Nrf2), a pivotal transcription factor responsive to ROS oxidative stress, orchestrates the transcriptional activation of genes involved in antioxidants, antioxidant biosynthesis, and metabolic flux transitions [24]. Under normal nonstress conditions, Nrf2 undergoes ubiquitination by the Keap1-E3 ubiquitin-ligase complex, involving Cullin 3, and subsequent degradation by the 26 S proteasome. Upon the mitohormic impact of ROS, mitochondrial ROS induce Nrf2 phosphorylation by PKCδ, facilitating its translocation into the nucleus [25]. Additionally, ROS prompt the relocation of Mst1/2 from the cytoplasm to mitochondria. Keap1 is translocated to mitochondria and phosphorylated by Mst1/2 at four Ser/Thr residues at its N-terminus, leading to Keap1 dimerization and subsequent release of Nrf2 [26, 27]. Hence, these findings imply that mitochondrial ROS activate Nrf2 in an indirect and kinase-dependent manner. Subsequent to gene induction in the nucleus, Nrf2 undergoes phosphorylation by Fyn, a member of the SRC family of tyrosine kinases, culminating in ubiquitination and degradation of Nrf2 [28]. Nrf2 orchestrates the regulation of a cadre of cytoprotective enzymes, including heme oxygenase-1 (HO-1), NAD(P)H-quinone oxidoreductase (NQO1), thioredoxin, peroxiredoxin, and glutathione peroxidase, via the antioxidant response element (ARE), in response to diverse oxidative stresses [29]. Furthermore, ROS-activated Nrf2 amplifies the expression of nuclear respiratory factor 1/2 (NRF-1/2), PGC-1α, and mitochondrial transcription factor A (TFAM), thereby promoting mitochondrial biogenesis [30]. Evidence indicates direct binding of Nrf2 to the promoter region of PINK1 [31]. The mitohormetic effect on promoting fission appears logical, as fission facilitates the removal of damaged mitochondria through mitophagy and enhances mitochondrial membrane potential [32]. However, ROS may induce mitophagy by disrupting mitochondrial membrane potential, enabling the recruitment of PINK1 to the mitochondrial outer membrane. The membrane-bound PINK1 protein stabilizes and undergoes autophosphorylation, initiating the recruitment of Parkin, a soluble E3 ubiquitin ligase. Parkin ubiquitinates various mitochondrial outer proteins, culminating in subsequent autophagy, phagocytosis, and lysosomal degradation [33]. Recent investigations emphasize the PINK1/Parkin pathway’s involvement solely in stress-activated mitophagy in a mitochondrial potential-dependent manner [34]. Nevertheless, some studies propose that the Keap1-Nrf2 stress response pathway diminishes mitochondrial fission and encourages excessive mitochondrial fusion by degrading the mitochondrial fission protein Drp1 [35]. This dynamic shift results in an over fused mitochondrial network with potential cellular protective effects against oxidative stress. In summary, we posit that slightly elevated ROS establish a positive feedback loop influencing mitochondrial kinetics and mitophagy. ROS can induce mitochondrial dynamic changes by disrupting the membrane potential and regulate PINK1/Parkin-mediated mitophagy activity in an Nrf2-dependent manner, thereby achieving mitochondrial quality control.

Beyond Nrf2, several other key regulators, including PGC-1α, FOXO3a, HSF, and NFκB, assume crucial roles in ROS-induced mitohormesis. Activation of PGC-1α occurs through AMPK or p38-MAPK phosphorylation [36, 37] and sirtuin 1 (SIRT1)-mediated deacetylation [38], markedly amplifying the transcription of proteins vital for mitochondrial biogenesis and respiration. Noteworthy is the finding that sestrin2, a stress-inducing protein in brown adipose tissue, fosters mitochondrial biogenesis by activating AMPK and subsequently enhancing PGC-1α activity. Sestrin2 also exhibits antioxidant functions when stimulated by ROS at non-toxic levels [39]. FOXO3a, akin to PGC-1α, undergoes activation via AMPK phosphorylation [40] and deacetylation mediated by SIRT1 [41] and SIRT3 [42]. While FOXO3a augments TFAM expression, its predominant role lies in enhancing the expression of proteins endowed with antioxidant and repair functions. Additionally, HSF1 and NFκB are acknowledged as downstream effector molecules in ROS-induced mitohormetic effects [43, 44]. However, investigations in this realm are currently limited, and the involvement of the HSF signal regulated by ROS may be partially linked to the unfolded protein response (UPR), necessitating further exploration.

## UPRmt

**Fig. 3:**
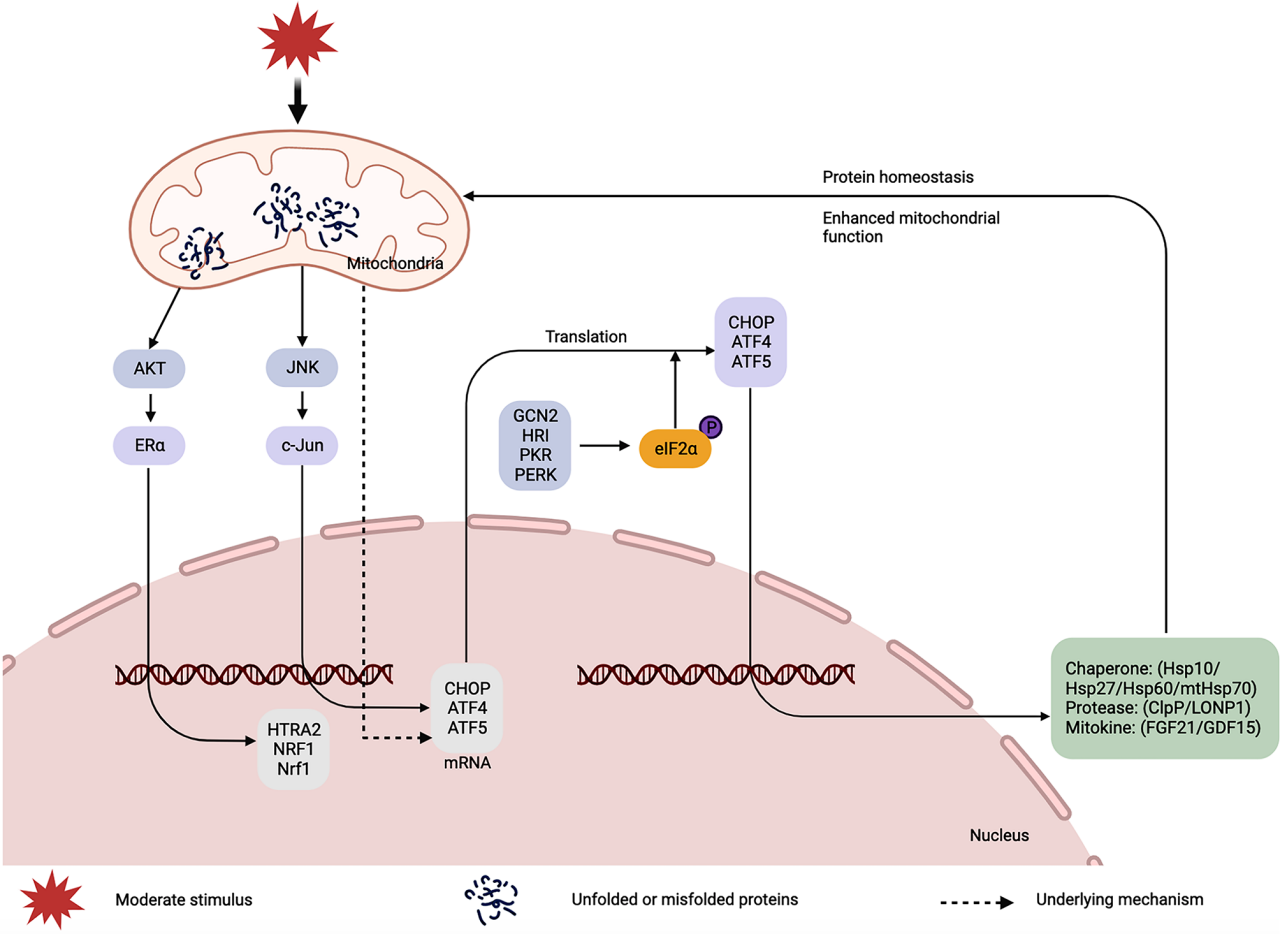
Mechanism of mitochondrial unfolded protein reaction (UPRmt) in mitohormesis. c-Jun: jun proto-oncogene, ClpP: caseinolytic protease P, eIF2α: eukaryotic translation initiation factor 2, Erα: estrogen receptor alpha, FGF21: fibroblast growth factor 21, GCN2: general control nonderepressible 2, GDF15: growth differentiation factor 15, HRI: heme-regulated inhibitor, Hsp: heat shock protein, HTRA2: high temperature requirement protein A2, JNK: c-Jun N-terminal kinase, LONP1: Lon protease 1, NRF1: nuclear respiratory factor1, Nrf1: NF-E2 p45-related factor 1, PERK: PKR‐like endoplasmic reticulum kinase, Perk: PKR‐like endoplasmic reticulum kinase, PKR: protein kinase R

Mild stimulation-induced protein imbalance triggers adaptive responses within mitochondria, activating distinct mitochondrial cytoplasmic and mitochondrial nuclear stress reactions. These responses play a pivotal role in regulating gene transcription, translation, posttranslational modification, and protein degradation to sustain protein stability, as depicted in Fig. 3. The UPRmt predominantly hinges on the subsequent regulation of three bZIP transcription factors, namely CHOP, ATF4, and ATF5, to facilitate the degradation of unfolded or misfolded proteins by mitochondrial chaperone proteins (Hsp10/Hsp27/Hsp60/mtHsp70) and proteases (ClpP/LONP1), thereby reconstructing mitochondrial protein homeostasis [18, 45–47]. Throughout the mitochondrial stress response, the activation of UPRmt predominantly relies on the transcription factors ATF4 and ATF5, which typically accumulate and degrade within mitochondria under physiological conditions. In response to stimulation, ATF5 translocates from mitochondria to the nucleus, thus regulating downstream protein expression [48–50]. Current understanding posits that the expression of these three transcription factors necessitates eIF2α phosphorylation [51, 52]. The sensing of stresses involves four kinases—GCN2, HRI, PKR, and PERK—which collectively contribute to the phosphorylation of a specific serine on the eukaryotic translation initiation factor eIF2α [53]. Furthermore, Haynes et al. postulated that the combination of CHOP, ATF4, and eIF2α phosphorylation is adequate to induce ATF5 expression [46]. Notably, c-jun and JNK have been identified as the upstream mechanism in the UPRmt signaling system, responsible for regulating CHOP. Upon JNK activation, the transcription factor c-Jun binds to the activator protein-1 (AP-1) consensus sequence, thereby promoting CHOP expression [54, 55]. It is crucial to highlight that, aside from CHOP, ATF4, and ATF5, UPRmt activation necessitates the involvement of the nuclear receptor ERα when disturbances occur within the mitochondria intermembrane space (IMS) [56]. ERα-mediated signaling pathways are activated through AKT phosphorylation, leading to increased expression of the IMS protease HTRA2. This, in turn, eliminates dislocated or defective IMS proteins and concurrently upregulates the expression of transcription factors NRF1 and Nrf1. NRF1 and Nrf1 play distinct roles in promoting mitochondrial biogenesis and protease expression, respectively [7, 46, 56, 57].

## MDP

**Fig. 4:**
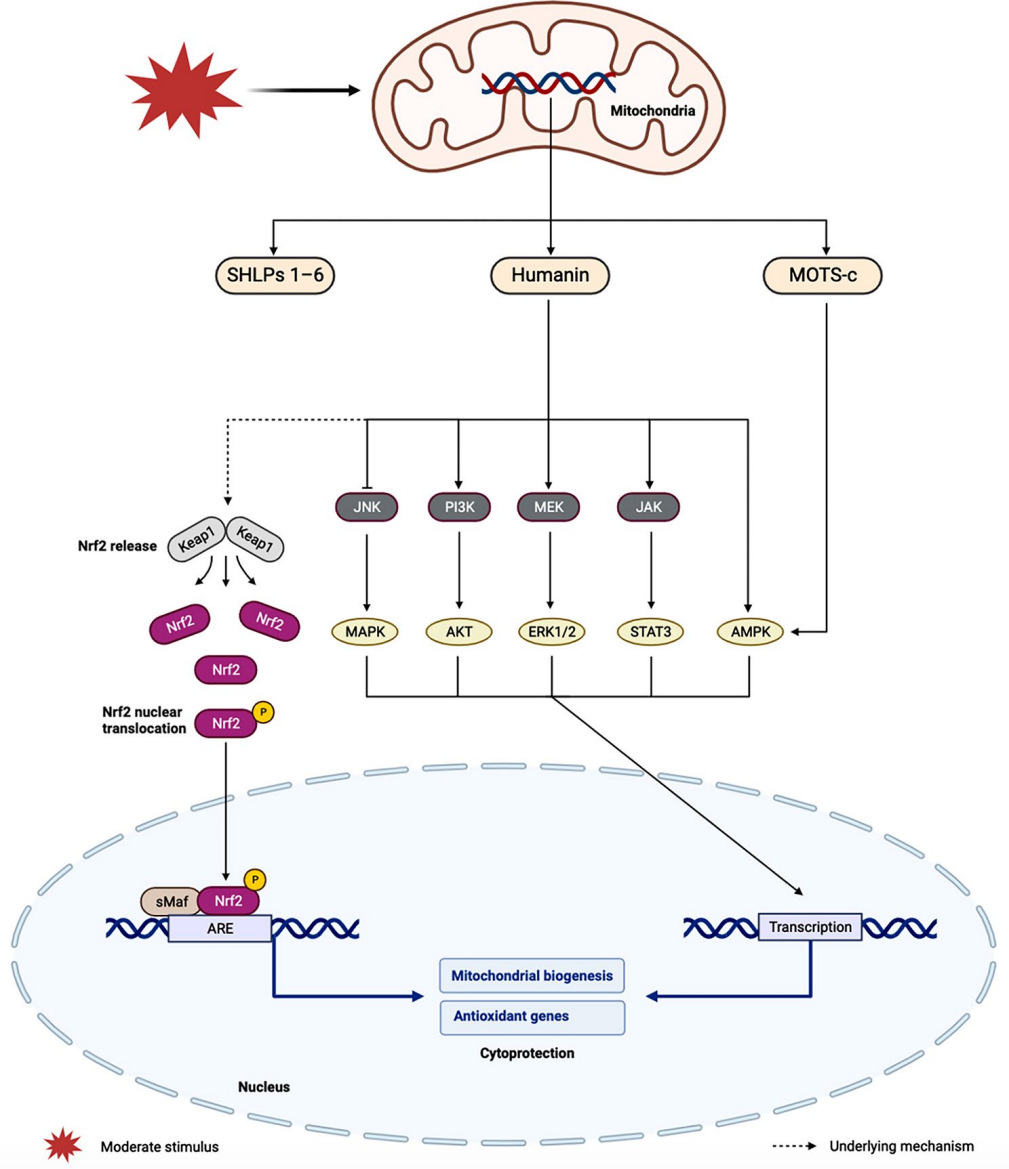
Schematic illustration of mitochondrial derived peptide (MDP) regulation of mitohormesis. ERK1/2: extracellular signal-related kinases 1 and 2, JAK: janus kinase, MEK: mitogen-activated extracellular signal-regulated kinase, MOTS-c: mitochondrial ORF of the twelve S c, PI3K: phosphoinositide 3-kinase, STAT3: signal transducer and activator of transcription 3

MDP refer to short bioactive peptides (< 100–150 amino acids) encoded by the short open reading frame of mitochondrial DNA. Notable examples include humanin, MOTS-c, and SHLPs 1–6 [58], which cells express in response to repeated stressor stimuli to activate mitohormesis, as illustrated in Fig. 4. Through the mitochondria-nuclear retrograde signal transduction pathway, mitochondria possess the capability to regulate the expression of nuclear genes. Humanin, among these MDPs, plays a pivotal role in mediating a diverse array of intracellular and extracellular signaling pathways, exerting multiple cellular protective functions. Wang et al. suggested that humanin may induce the degradation of Keap1 and the subsequent release of Nrf2, thereby promoting antioxidant gene expression and facilitating the recovery of mitochondrial function [59]. Humanin induces the phosphorylation of AMPK, contributing to the induction of PGC-1α, NRF1, and TFAM, thereby significantly enhancing mitochondrial biogenesis [60]. Furthermore, Humanin elevates the phosphorylation levels of AKT, ERK1/2, and STAT3, with the involvement of PI3K, MEK, and JAK in the activation of these signaling pathways [61]. This activation, in turn, promotes the expression of antioxidant proteins, resulting in reduced ROS levels and mitigated oxidative stress damage. Humanin also exerts an inhibitory effect on calcium overload, consequently suppressing MAPK (JNK/p38) signaling, and enhancing mitochondrial oxidative phosphorylation and biosynthesis [62]. Notably, Humanin activates autophagy signaling mediated by Hsp90, playing a crucial role in stress resistance [63]. MOTS-c, another mitochondrial polypeptide, assumes a pivotal role in the retrograde signal transduction pathway [64]. In an AMPK-dependent manner, MOTS-c translocates to the nucleus, where it interacts with ARE-regulating stress-responsive transfer factors, including Nrf2, thereby enhancing cellular stress resistance [65, 66]. MOTS-c further contributes to metabolic reprogramming by regulating downstream effectors such as HSF1 and HO-1, ultimately restoring mitochondrial function [58, 67]. MOTS-c inhibits oophorectomy-induced bone loss through AMPK activation [68].

In a broader context, mitochondrial-derived peptides encompass not only peptides encoded by mitochondrial DNA but also cytokines encoded by nuclear genes. These cytokines are induced when mitochondria are stimulated by stress, acting as endocrine or paracrine factors. FGF21 and GDF15 stand out as the two pivotal factors in mitohormesis, regulating the adaptive responses of the entire body [69, 70]. The upregulation and secretion of FGF21 have been demonstrated to depend on PI3K/AKT signaling [71]. The autocrine activity of FGF21 induces the upregulation of PGC-1α, enhancing mitochondrial oxidation function by activating AMPK and SIRT1 [72, 73]. GDF15, categorized as a stress response cytokine of the TGF-β superfamily, is regulated by the transcription factor CHOP in the UPRmt pathway. CHOP can directly bind to the promoter of GDF15 and activate its transcription under stress conditions [74]. The mitohormesis effect results from the synergistic action of multiple networks, where signal transduction and positive feedback loops between mitochondria and the nucleus collaboratively enhance the stress resistance of cells.

## Mitochondrial dynamic changes and mitophagy

Mitohormesis is contingent upon alterations in mitochondrial dynamics and mitochondrial autophagy. Mitochondrial dynamics, involving fission and fusion processes, plays a crucial role in maintaining mitochondrial function and morphology [75]. Mitochondrial fission relies on the induction of cytoplasmic dynamics-related proteins (Drp1 and Drp2) and FIS1 located in the outer membrane, while mitochondrial fusion is mediated by Mfn1 and Mfn2 in the outer membrane and OPA1 in the inner membrane [76]. The elimination of defective mitochondria is ensured through fission, fusion, and mitophagy, aiming to retain mitochondria with complete mitochondrial DNA copy number, membrane composition, and optimal metabolic capacity [21, 77, 78].

Under stress stimulation, the inner membrane depolarizes, leading to the accumulation of PINK1 in the outer membrane, promoting Parkin recruitment to the mitochondrial surface and inducing autophagy for the clearance of damaged mitochondria [21]. Despite potential oxidative stress-induced mutations in mitochondrial DNA (mtDNA), mtDNA with mutations may still fuse with other mitochondria in the same cell through Mfn2 mediation when the mutation load is mild [19, 79]. Additionally, migrasome-mediated mitochondrial exocytosis plays a crucial role in cases of mild mitochondrial damage, assisting in the progress of mitohormesis. During mitochondrial exocytosis, damaged mitochondria are transported outward by the motor protein kinesin family member 5B (KIF5B) and subsequently attached to cortactin by myosin [20]. Following Drp1-mediated mitochondrial division, dysfunctional mitochondria are packaged as migrasomes and excluded from the cell [80]. Studies by Zhang et al. indicate that moderate mechanical stress stimulation enhances the efficiency of mitochondrial dynamics conversion by upregulating the expression of Mfn1/2, OPA1, and the translocation of Drp1 from the cytoplasm to mitochondria [81]. Similarly, Yao et al. observed an upregulation in the expression of Mfn2, FIS1, and Parkin within 24 h in IL-1β-treated chondrocytes, speculating that short-term IL‐1β stimulation promotes mitochondrial dynamics activity and mitophagy [82]. Mitophagy functions to engulf selectively separated mitochondrial fragments resulting from fusion and fission processes, contributing to the reconstruction of mitochondrial homeostasis.

Therefore, the equilibrium between mitochondrial dynamic changes and mitophagy activity stands as the pivotal determinant of mitochondrial structure and function. In essence, mitohormesis predominantly relies on a convergence of multiple signaling pathways, encompassing ROS, metabolites, UPRmt, mitochondrial-cytoplasmic stress responses, MDP secretion, and mitochondrial structural alterations.

### Mitochondrial dysfunction is involved in aging-related bone and cartilage degeneration

Within the intracellular microenvironment, mitochondrial dysfunction stands out as a central mechanism in cellular aging injury. Anomalies in oxidative phosphorylation result in reduced ATP synthesis and stimulate the generation of excessive ROS. Oxidative stress injury is regarded as the paramount pathogenic factor associated with cellular senescence phenotypes observed in OA, IVDD, and OP [83–85]. The mechanism of cell damage induced by ROS exhibits a high degree of uniformity, primarily involving two aspects: direct oxidative damage to lipids, proteins, and DNA; and participation in signal cascade amplification as a second messenger [86].

OA is a gradual synovial joint disease characterized by focal articular cartilage destruction and a hypertrophic response in adjacent bone. This process results in subchondral bone remodeling, osteophyte formation, and varying degrees of synovial inflammation, joint capsule thickening, and structural destruction of soft tissues [87]. Predominantly, cartilage degeneration is ascribed to extracellular matrix abnormalities, encompassing the deposition of advanced glycosylation end products, proteoglycan deficiency, and collagen hydrolysis [88]. Aging and mechanical overload are recognized as the most significant risk factors for OA [89, 90]. The aging microenvironment of osteoarthritis extends beyond chondrocytes to include synovial fibroblasts and synovial macrophages [91].

IVDD is characterized by dehydration of the nucleus pulposus, annulus fibrosus tear, and loss of extracellular matrix components such as collagen fibers and proteoglycan [92]. Aging emerges as a major risk factor for IVDD development [93]. The interplay of aging and continuous mechanical stress disrupts intervertebral disc metabolism, resulting in an imbalance between the expression of catabolic factors (e.g., proinflammatory cytokines and matrix metalloproteinases) and anabolic molecules (e.g., growth factors). This imbalance ultimately confuses extracellular matrix homeostasis, promoting intervertebral disc degeneration [94, 95]. The aging microenvironment of intervertebral discs encompasses nucleus pulposus cells, annulus fibrosus cells, and chondrocytes [96].

OP is characterized by decreased bone density, microstructure destruction of bone tissue, increased bone fragility, and susceptibility to fractures. The fundamental pathological mechanism of osteoporosis involves an imbalance in bone remodeling homeostasis between osteoblast bone formation and osteoclast bone resorption [97]. Aging and a lack of mechanical stimulation are closely linked to the occurrence and development of osteoporosis [98–100]. The aging microenvironments of osteoporosis encompass bone marrow mesenchymal stem cells (BMSCs), osteoblasts, osteoclasts, and osteocytes [5].

Excessive ROS instigate lipid peroxidation and glycosylation, leading to an augmented production of endogenous active aldehydes and their byproducts (glyoxal, methyl glyoxal, malondialdehyde, and 4-hydroxy-2-nonenal), advanced lipoxidation end products (ALE), and advanced glycation end products (AGE) [101]. Elevated levels of ALE and AGE serve as robust indicators of age-related oxidative damage in cartilage and intervertebral discs, potentially hastening the degeneration process by promoting apoptosis and inhibiting extracellular matrix metabolism [102–105]. Notably, the levels of carbonyl protein and advanced oxidation protein products (AOPPs) in degenerative intervertebral discs exhibit a significant increase [106]. Proteins within the discs of older mice manifest a higher content of oxidized amino acids compared to those of younger mice [106, 107].Moreover, oxidative modification of intervertebral disc collagen induces collagen crosslinking and aggregation, leading to conformational changes in proteins that result in collagen cleavage. This ultimately impacts the anatomical integrity and biomechanical properties of the intervertebral disc [107–109]. ROS-induced chondrocyte apoptosis occurs through the reduction of mtDNA integrity and repair capability due to oxidative damage [110]. MtDNA damage and mutation yield respiratory chain subunits with impaired mitochondrial function, hindering effective chondrocyte differentiation. This, in turn, exacerbates ROS production and promotes apoptosis [111]. Similarly, DNA damage induced by ROS in nucleus pulposus cells and osteoblasts has been observed in age-related disc degeneration and osteoporosis [112, 113].Furthermore, ROS regulate cell signaling through the posttranslational oxidative modification of specific mercaptan groups in proteins containing active cysteine [114]. ROS induce sulfonylation of various chondrocyte proteins, including tyrosine kinase (SRC), thereby promoting the expression of matrix metalloproteinase 13 (MMP-13) [115]. These findings suggest that ROS-induced sulfonylation of chondrocyte proteins may activate signaling pathways contributing to cartilage matrix degradation. Additionally, aging-associated ROS-induced hyperoxidation of cysteine reduces the activity of redox-sensitive proteins, ultimately leading to chondrocyte death [116].

Studies have demonstrated that oxidative stress in the mitochondria of aged chondrocytes differentially inhibits the phosphorylation of AKT mediated by insulin-like growth factor-1 (IGF-1) while activating the MEK-ERK MAPK signaling pathway. This alteration affects chondrocyte metabolism, resulting in a decrease in extracellular matrix gene expression and protein synthesis [117, 118]. ROS can activate various signaling pathways, including the p38-MAPK, ERK, and JNK pathways, triggering nuclear translocation of NFκB and Nrf2. This activation promotes the senescence phenotype in chondrocytes, nucleus pulposus cells, and osteoblasts [119–121]. Mitochondrial dysfunction and ROS production further stimulate the release of cytokines involved in the regulation of aging-related secretory phenotypes through mechanisms such as the activation of IL-1, IL-6, and inflammasomes assembled from NOD-like receptor family pyrin domain-containing 3 (NLRP3). These processes exacerbate apoptosis and contribute to the development of osteoarthritis and disc degeneration [122–125]. It is evident from the above that oxidative stress damage caused by mitochondrial dysfunction in senescent cells establishes a positive feedback loop, intensifying the senescence-related phenotype and promoting the progression of bone and cartilage degeneration.

## Activation of mitohormesis to combat degenerative changes in bone and cartilage

**Fig. 5:**
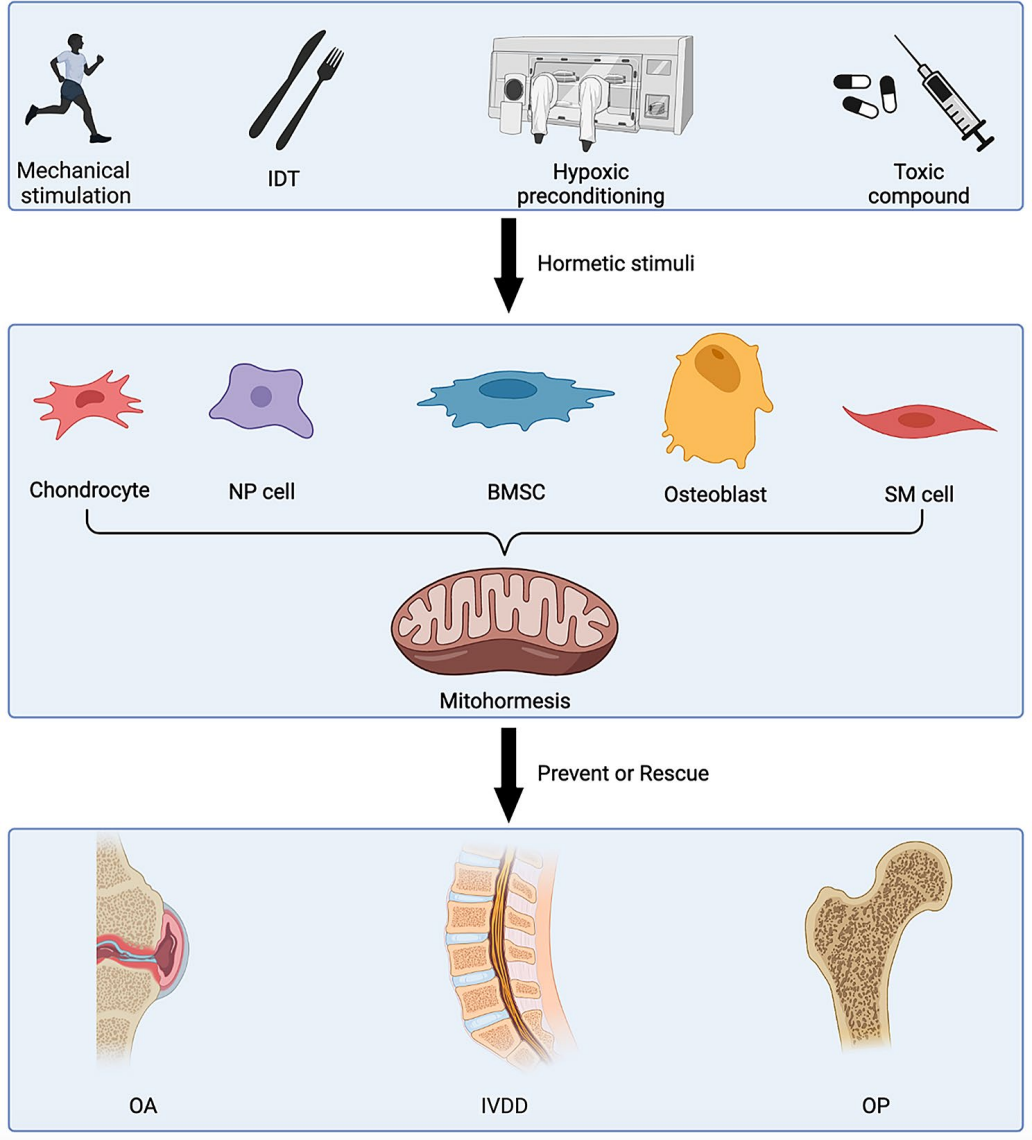
Activation of mitohormesis to relieve bone and cartilage degeneration. IDT: intermittent dietary restriction, IVD: intervertebral disc degeneration, NP: nucleus pulposus, OA: osteoarthritis, OP: osteoporosis, SM; smooth muscle

Aging-related degenerative diseases affecting bone and cartilage entail prolonged chronic progression, with the diminished vitality and stress resistance of cells in the bone microenvironment representing key pathogenic factors. Illustrated in Fig. 5 are current stimuli with the potential to augment the physiological anti-damage mechanism and impede the progression of these degenerative diseases by activating mitohormesis effects. These stimuli encompass mechanical stimulation, intermittent dietary restriction, hypoxic preconditioning, and exposure to certain toxic compounds.

## Mechanical stimulation

The fundamental characteristics of cells in the bone and cartilage microenvironment include sensitivity and an adaptive response to mechanical stimulation [126]. Exercise serves as an effective stimulus for inducing mitohormesis [11, 127]. Varied intensity, duration, and frequency of exercise prompt diverse mitochondrial changes, encompassing biogenesis, oxidative stress, UPRmt, secretion of MDP, fusion and fission, and mitophagy [128, 129]. Physiological-level mechanical stimulation can sustain normal energy metabolism and intracellular homeostasis. It achieves this by inducing moderate ROS stress and the UPRmt response through mitochondria-dependent pathways, ultimately enhancing cell tolerance [130]. When chondrocytes experience physiological circulatory loads, mitochondria generate beneficial levels of ROS that support metabolism, homeostasis, and matrix synthesis [131, 132].

Research indicates that various forms of exercise, including nontraditional activities like tai chi and yoga, can mitigate degenerative injuries, enhance pain relief, improve limb function, and elevate the quality of life in osteoarthritis patients [133]. Notably, low-intensity activity significantly inhibits the onset and progression of osteoarthritis [134]. For instance, walking can prevent subchondral cyst formation, cartilage degeneration, and inhibit osteoclast activity and osteocyte apoptosis [135]. Moderate-intensity exercise hinders cartilage degeneration, extracellular matrix loss, chondrocyte apoptosis, inflammation, and ROS-mediated positive feedback loops [136]. Furthermore, exercise effectively improves bone mass and strength in senile osteoporosis [137, 138]. Dynamic mechanical loading and sufficiently high strain (> 5 microstrain) are essential for inducing bone remodeling [136, 139]. Recent studies have demonstrated that high-intensity progressive resistance exercise enhances bone mass in older individuals [140–142]. Niehoff et al. found that exercise protects the bones of mice deficient in superoxide dismutase (SOD2) from oxidative stress [143]. Similarly, tai chi and other exercises can reduce oxidative stress levels in postmenopausal women with osteopenia [144]. However, research on intervertebral disc degeneration has primarily focused on direct exogenous antioxidant interventions, with limited evidence demonstrating exercise’s regulation of ROS levels to activate mitohormesis in nucleus pulposus cells. In conclusion, the interplay between exercise, oxidative stress, and mitochondrial function regulation is complex in the intervertebral disc microenvironment, with no strict criteria for determining the intensity and duration of motor and mechanical stimuli. Excessive mechanical stimulation can cause stress damage that surpasses mitochondrial adaptive flux, leading to dysfunction. Conversely, moderate exercise stimulation may enhance antioxidant response efficiency by activating mitohormesis.

In conclusion, our contention is that ROS, stemming from mitochondrial dysfunction, escalate with age. Excessive ROS levels induce damage to chondrocytes, rendering them more susceptible to the aging process. Furthermore, an excess accumulation of ROS within the bone marrow niche alters the presentation of cell signaling cascades, impedes the osteogenic differentiation of BMSCs, and amplifies osteoclast activity. Mechanical stimulation has the capacity to directly engage cells in exercise, stimulating the mitohormesis effect. This, in turn, prevents ROS-mediated crosstalk between cells and ultimately manifests a protective effect on bone and cartilage.

## Intermittent dietary restriction (IDT)

Limiting calorie intake to 60–70% of normal adult weight maintenance requirements has been demonstrated to extend life by 30–50% and confer nearly optimal health [145]. Ketone bodies, generated through the β-oxidation of fatty acids and released as supplementary fuel during intermittent dietary restriction (IDT), induce oxidative stress that, paradoxically, offers long-term benefits by triggering hormetic responses. Initially, ketone bodies stimulate the mitochondrial production of ROS, activating key regulators of cellular protective mechanisms, including Nrf2, AMPK, SIRT1, PGC-1α, and FOXO3a. This activation enhances antioxidant activity, DNA repair, and autophagy [12, 146].

IDT has exhibited noteworthy health benefits, including a discernible impact on osteoarthritis, manifesting within a brief two-week period [145, 147]. Clinical investigations have discerned that ketogenic diets exert a significant improvement in both knee pain and functional outcomes among patients grappling with type 2 diabetes [148]. In a study conducted by Stephen et al., where the effects of dietary restriction and/or exercise were scrutinized in a cohort of 450 knee osteoarthritis patients, it was observed that the combination of IDT and exercise yielded markedly superior positive effects compared to exercise in isolation [149]. The documented influence of IDT extends to pivotal aspects such as inflammation, insulin resistance, and cardiovascular disease. Furthermore, there is suggestive evidence that IDT may enhance bone health by mitigating recognized risk factors associated with osteoporosis. A study investigating the effects of fasting during Ramadan on bone metabolism concluded that dietary practices characteristic of Muslim festivals may confer benefits for bone turnover [150, 151]. The imperative for well-designed studies to thoroughly elucidate the role of IDT in bone metabolism is underscored.

Currently, numerous published studies have explored the favorable outcomes of IDT in the context of bone and cartilage degenerative diseases. The majority of these studies have predominantly focused on systemic regulation, specifically in terms of reducing obesity and inflammation. However, limited research has investigated the mechanistic aspects of IDT in terms of its impact on cellular mitochondrial metabolism. Our hypothesis posits that the activated mitochondrial hormesis effect serves as the fundamental mechanism underpinning the protective effects of IDT on bone and cartilage. Subsequent studies should aim to delineate the intricacies of cell-specific and species-specific nuances in the personalized design of IDT protocols.

## Hypoxic preconditioning

An extreme anoxic oxygen tension of 0.5% leads to heightened oxidative stress and cellular apoptosis [152]. In contrast, mild hypoxia within the range of 0.5–3% O_2_ prompts the release of mitochondrial ROS that function as signaling messengers, initiating various biological processes and mitohormesis. This includes the activation of the transcription factor hypoxia-inducible factor (HIF), which supports the survival of stem cells in hypoxic conditions [7]. Mesenchymal stem cells (MSCs) cultured under 1% O_2_ hypoxic conditions for approximately 2 weeks exhibit enhanced proliferation and viability, thereby retarding phenotypic changes such as increased cell size, altered morphology, and the expression of senescence-associated β-galactosidase [153].

Physiologically, the microenvironments of bone and cartilage are naturally exposed to hypoxia, with levels ranging from 1% in cartilage to 1–7% in bone marrow. The activated HIF-1α signal in response to hypoxia enhances glycogen storage, preventing energy deficiency and contributing to cell survival. Pathological disruption of the HIF cascade is implicated in bone and cartilage degenerative diseases [154–156]. Hence, even a slight decrease in ambient oxygen concentration or the use of hypoxia simulators activates downstream target genes, initiating adaptive responses to hypoxia. Hypoxia preconditioning activates the mitohormesis effect in bone and cartilage microenvironment cells, enhancing stress resistance and cellular activity. Under 1% O_2_ hypoxic conditions, mouse chondrocytes showed increased HIF expression, triggering a stable autophagy protection mechanism that prevents mitochondrial dysfunction, apoptosis, and senescence, thereby improving cartilage degradation in OA mice [157]. Chronic intermittent hypoxia preconditioning (simulating 3000 m altitude, 5 h/day for 28 days, PO_2_ = 108.8 mmHg) demonstrated protective effects against collagen-induced osteoarthritis in rats [158]. In addition, Idrus et al. comprehensively analyzed studies on OA treatment involving the creation of anoxic microenvironments [159]. Current research on osteoporosis targets HIF signal activation or inhibition of HIF protein degradation (using deferoxamine, cobalt chloride, and dimethyloxaloylglycine) to simulate hypoxic conditions for therapeutic effects [160, 161]. Furthermore, MSCs cultured with 1% O_2_ for 7 days exhibited increased proliferation, migration, and promoted osteogenic differentiation [162]. Periodic hypoxia (3% O_2_ for 1, 2, or 4 h, 4 days/week) regulates MSC differentiation, improving bone health in aging [163]. A randomized clinical trial found that 24 weeks of normobaric cyclic hypoxic exposure combined with resistance circuit training could potentially yield positive effects on bone in older individuals [164]. Timon et al. discovered that 18 weeks of whole-body vibration training with hypoxic stimuli positively affected bone density in elderly individuals [165]. The combination of moderate mechanical stimulation with intermittent hypoxia treatment demonstrated significant benefits on bone metabolism. The nucleus pulposus, characterized by an anoxic and avascular environment, is protected against intervertebral disc degeneration through chronic periodic hypoxic preconditioning (3000 m altitude, 5 h/day for 28 days, PO_2_ = 108.8 mmHg) in rats [166]. Do et al. assessed phenotype changes in nucleus pulposus cells at two oxygen tension levels (5% and 20%) up to serial passage 20, finding that hypoxia significantly increased autophagosome numbers, activating autophagic flux and concurrently reducing apoptotic protein expression to maintain nucleus homeostasis [167]. In conclusion, hypoxia proves beneficial in delaying the progression of bone and cartilage degenerative diseases, but a deeper exploration of the underlying mechanisms between hypoxia and mitohormesis is warranted.

## Toxic compounds

Toxic compounds do not invariably result in pathological outcomes, as mitochondria can function as an adaptive mechanism, inducing diverse stress responses, particularly when stressors are transient. In fact, exposure to mitochondrial toxins, causing transient mitochondrial stress, can be beneficial by fostering mitochondrial hormesis [168]. Resveratrol, a small-molecule compound, effectively emulates the impacts of dietary restrictions. The chronic intermittent application of resveratrol (10 µM) not only enhances bone formation and mitigates accelerated bone loss but also improves osteogenic differentiation in aged BMSCs by stimulating mitochondrial autonomous gene transcription and facilitating mitochondrial functional recovery [169]. Moreover, rotenone, an inhibitor of the electron transport chain, seems to replicate the effects of hypoxic preconditioning. Low concentrations of rotenone (12.5 nM) may induce mitohormesis by modestly elevating ROS levels, thereby increasing SIRT1 expression and subsequent deacetylation of PGC-1α [170]. Additionally, various compounds, including As_2_O_3,_ arsenite, N-acetyloxyfenicine, 3-bromopyruvate, N-acetylcysteine, and L-lactate, at low concentrations, have demonstrated the capacity to induce mitohormesis in cells. All these compounds have shown that instantaneous ROS production improves the stress resistance of cells [171–176]. However, research on the preventive or therapeutic potential of low-dose toxic compounds that trigger mitohormesis in OA, IVDD, and OP remains limited.

## Future perspectives

- The excitatory impact of ROS on mitochondrial hormesis lacks accurate quantitative studies. Many investigations primarily describe the appropriate quantity, medium, and moderation, providing insufficient guidance for subsequent in-depth examinations of exogenous interventions to activate mitochondrial hormesis.
- The influence of ROS on stress resistance relies not only on ROS concentration but also on their cellular localization. Notably, increased mitochondrial ROS levels significantly enhance cell longevity and stress resistance, while elevated cytoplasmic ROS levels have the opposite effect.
- Exploring hypoxia preconditioning as a research direction to enhance cellular anti-stress capabilities holds promise. The low perfusion characteristics of bone and cartilage microenvironments introduce complexity to the relationship between hypoxia preconditioning and the enhancement of mitochondrial function.
- Dietary restrictions exhibit the potential to decelerate the progression of aging-related diseases. It is imperative to investigate, at the cellular level, the role of nutritional restriction in mitigating bone and cartilage degeneration. However, the impact of dietary restrictions on bone and cartilage health is contentious, with specific dietary restrictions (type, proportion, and duration of food intake) becoming the focal point for future research.
- The synergistic effects of mechanisms in mitohormesis, signal coupling, and positive feedback loops involving ROS, mitochondrial UPRmt, MDP, mitochondrial dynamics, and mitophagy require further elucidation.

## Conclusion

Mitochondrial hormesis, serving as a cellular adaptive mechanism to diverse stressors, has garnered significant attention in unraveling disease pathogenesis. In recent years, scholars have delved into the therapeutic potential of mitochondrial hormesis, particularly in cardiovascular and neurodegenerative diseases. The degenerative changes in bone and cartilage associated with aging are intricately linked to mitochondrial dysfunction. However, prevailing studies predominantly concentrate on the interplay between mitochondrial hormesis induced by skeletal muscle exercise and aging. Notably, there is a dearth of research addressing direct stimulation to instigate mitohormetic effects and restore the functionality of chondrocytes, nucleus pulposus cells, BMSCs, and osteoblasts. In the broader physiological context, bone and muscle exhibit systemic regulation. Age-related muscle loss significantly correlates with the onset and progression of OA, IVDD, and OP. ROS production in skeletal muscle cells, arising as a byproduct of mitochondrial oxidative phosphorylation, predominantly governs mitochondrial hormesis during exercise or muscle contraction. Presently, aerobic exercise stands as a partial remedy for age-related muscle decline, with moderate exercise potentially mitigating the advancement of age-related degenerative bone and cartilage conditions by enhancing muscle quality and function.

## Data Availability

The data that support the findings of this study are available from the corresponding author upon reasonable request.

## Abbreviations

AGE: advanced glycation end products
ALE: advanced lipoxidation end products
AOPPs: advanced oxidation protein products
ARE: antioxidant response element
FGF21: fibroblast growth factor 21
GDF15: growth differentiation factor 15
HIF: hypoxia-inducible factor
HO-1: heme oxygenase-1
IDT: intermittent dietary restriction
IGF-1: insulin-like growth factor-1
IVDD: intervertebral disc degeneration
KIF5B: motor protein kinesin family member 5B
MDP: mitochondrial-derived peptides
NLRP3: NOD-like receptor family pyrin domain-containing 3
NQO1: NAD(P)H-quinone oxidoreductase
NRF-1/2: nuclear respiratory factor 1/2
Nrf2: nuclear factor (erythroid-derived 2)-like 2
OA: osteoarthritis
OP: osteoporosis
ROS: reactive oxygen species
TFAM: mitochondrial transcription factor A
UPRmt: mitochondrial unfolded protein reaction
